# The TLR7 agonist vesatolimod does not measurably induce SIV expression in macaques receiving combination antiretroviral therapy initiated during chronic infection

**DOI:** 10.1128/aac.01073-25

**Published:** 2025-10-07

**Authors:** Adrienne E. Swanstrom, Kelli Oswald, Randy Fast, Rebecca Shoemaker, James A. Thomas, Cathi Pyle, Michael Hull, William J. Bosche, Yuan Li, Matthew W. Breed, Joshua A. Kramer, Duncan Donohue, Charles M. Trubey, Claire Deleage, Romas Geleziunas, Jeffrey D. Lifson, Gregory Q. Del Prete

**Affiliations:** 1 AIDS and Cancer Virus Program, Frederick National Laboratory for Cancer Research 437329 , Frederick, Maryland, USA; 2 Laboratory Animal Sciences Program, Frederick National Laboratory for Cancer Research 437329 , Frederick, Maryland, USA; 3 Statistical Consulting and Scientific Programming Group, Computer and Statistical Services, Data Management Services, Inc, NCI-Frederick, Frederick, Maryland, USA; 4 Gilead Sciences 2158 , Foster City, California, USA; Chinese Academy of Medical Sciences & Peking Union Medical College, Beijing, China

**Keywords:** nonhuman primate, SIV, macaque, HIV latency, HIV cure, HIV-1

## Abstract

Lim et al. previously reported that TLR7 agonist (Vesatolimod [VES] or the related compound GS-986) administration to SIVmac251-infected macaques receiving combination antiretroviral therapy (cART) led to transient plasma viral load (PVL) increases, viral DNA (vDNA) reductions in blood and tissues, and, in some animals, extended viral remission after treatment cessation (S. Y. Lim, C. E. Osuna, P. T. Hraber, J. Hesselgesser, et al., Sci Transl Med 10:eaao4521, 2018, https://doi.org/10.1126/scitranslmed.aao4521). However, in multiple subsequent studies, TLR7 agonist administration in SIV or SHIV-infected macaques on cART did not induce measurable virus expression. Notably, these studies utilized earlier cART initiation, lengthier cART treatment before TLR7 agonist administration, different sampling time points, and/or less sensitive virologic assays compared to the Lim study. We hypothesized that study design and assay differences may have led to these apparently discrepant results due to quantitative or qualitative differences in the established viral reservoirs and/or a reduced capacity to detect virus induction. To more closely capture the Lim study conditions, we initiated cART at 65 days post-infection in 10 SIVmac239M-infected rhesus macaques. Six received VES (0.15 mg/kg orally, every 2 weeks for six doses), while four received vehicle control. Although VES treatment induced expected transient increases in interferon-stimulated gene expression and immune cell phenotypic changes, it did not lead to measurable PVL increases in peripheral or hepatic portal vein blood using a highly sensitive PVL assay or increases in cell-associated vRNA:vDNA ratios, nor to measurable reductions in vDNA in blood or tissues. These results align with recent clinical data and confirm that TLR7 agonist treatment does not reliably induce significant virus expression *in vivo*.

## INTRODUCTION

 During infection, HIV-1 establishes a rebound-competent viral reservoir (RCVR), a pool of long-lived sources of replication competent virus that can persist in the face of suppressive combination antiretroviral therapy (cART) for the lifetime of an individual with HIV (1–7). Most of the RCVR is composed of cells harboring transcriptionally silent, or latent, viral genomes (8–11), but in response to various triggers, a level of cellular activation leading to virus expression, up to and including the production of infectious virions may occur. Combination ART does not prevent this process, but rather it inhibits the *de novo* spread of infection to previously uninfected cells, thereby preventing progressive infection. However, if cART is interrupted, virus produced from one or more of these reactivating viral genomes can spread, leading to recrudescent systemic infection characterized by rebound plasma viral loads (PVL) (12–14) and, should cART not be resumed, eventual progression to AIDS. While the precise timing of RCVR formation has been the subject of debate, current data suggest that it forms early following initial infection and likely evolves over the course of untreated infection (15–19). Thus, due to the early establishment and longevity of the RCVR, lifelong cART initiated at first HIV diagnosis is now indicated for virtually all people with HIV (PWH). However, concerns about the costs, accessibility, lifelong compliance, and potential emergence of drug-resistant viral mutants make lifelong cART for the nearly 40 million PWH worldwide a daunting challenge. Additionally, PWH on cART still have an elevated risk of non-AIDS morbidities and mortality associated with persistently elevated immune activation and inflammation that may be due, in part, to the persistent presence of virally infected cells (20–24). Accordingly, there is a global research effort to identify more definitive treatment for HIV-1, be it true viral clearance or durable viral remission, that would allow for cART to be safely discontinued.

 While other sources of replication-competent virus may persist during cART, CD4+ T cells harboring intact viral genomes are the major constituent of the RCVR (25). At any given moment, the overwhelming majority of these viral genomes are latent (8–11), rendering most residual infected cells immunologically indistinguishable from uninfected cells, complicating efforts to target these cells for elimination using virus-specific approaches. Many proposed strategies to reduce the RCVR, therefore, involve an effort to induce viral expression from latent viral genomes, under cover of cART to prevent reactivating virus from infecting new cells, so that infected cells either will die due to viral cytopathicity or will express viral proteins that can be targeted by native or augmented viral clearance mechanisms. Though many suggested viral reactivation strategies have been evaluated *in vivo* in animal models and humans, most have been disappointing, with few notable exceptions (26). Moreover, the reversal of viral latency is just one hurdle to clear to effect meaningful RCVR reduction. Endogenous antiviral immune responses may be inadequate to effect substantial depletion of cells harboring reactivated viral genomes (27–30) and, therefore, proposed strategies to eliminate or reduce the RCVR typically aim to facilitate or enhance the immunologic targeting of cells expressing viral antigens in addition to viral reactivation. Accordingly, agents with the capacity to both reactivate viral gene expression and also enhance immune responses would be highly attractive for efforts to reduce or eradicate the RCVR.

 A previous study by Lim and coworkers suggested that stimulation of toll-like receptor 7 (TLR7) may provide this sought-after combination of viral reactivation and boosting of antiviral immune responses (31). The authors reported that repeated oral administration of the TLR7 agonist Vesatolimod (VES, GS-9620) or the related compound GS-986 to SIVmac251-infected rhesus macaques receiving combination antiretroviral therapy (cART) induced transient plasma viral load (PVL) increases in association with activation of both innate and adaptive immune cell populations, including CD4+ T cells (31). Provocatively, the authors also showed reductions in viral DNA in peripheral blood mononuclear cells (PBMC) and gut and lymph node tissues in animals treated with TLR7 agonists, suggestive of clearance of residual infected cells, with two of nine TLR7 agonist-treated animals showing no viral rebound upon cART cessation, indicative of infection remission (31). Taken together, the Lim et al. study data suggested that TLR7 agonist treatment simultaneously reactivated residual viral genomes and enhanced immunological clearance of cells harboring these genomes.

 In contrast to these promising results reported by Lim et al., and despite similar reported immunomodulatory effects, measurable increases in plasma viremia were not observed following TLR7 agonist administration to SIV- and SHIV-infected macaques on suppressive cART in a number of subsequent studies conducted by our group and others (32–39). However, there were key experimental design and analysis differences between the Lim study and these subsequent experiments. In the Lim study, cART was initiated during the chronic phase of infection, 65 days after SIV infection, with TLR7 agonist treatments beginning approximately 440 days after cART initiation (31). Plasma viral loads were monitored at 24 and 48 h after TLR7 agonist administration using an assay with a reported threshold sensitivity of 50 viral RNA (vRNA) copies/mL (31). By contrast, other published studies that involved TLR7 agonist administration to SIV/SHIV-infected macaques on cART utilized earlier cART initiation, during the acute phase of infection (32–34, 36–39), a longer duration of cART prior to TLR7 agonist treatment (33–35), and/or less sensitive virologic monitoring assays (32, 33, 38). Also, while the Lim study reported transient viremia detectable only within the first 24–48 h after TLR7 agonist administration, only two other studies similarly examined plasma viremia at both 24 and 48 h (32, 34), while the remaining studies assessed samples collected outside this time window or at unspecified time points (33, 35–39). Collectively, these differences in study design parameters and assay sensitivities raise the possibility that quantitative or qualitative differences in the established RCVR, and/or a reduced capacity to actually detect reactivating virus, may underlie these seemingly discrepant findings.

 To test the hypothesis that *in vivo* detection of potential TLR7 agonist-mediated virus induction is dependent upon the size or nature of the established viral reservoirs, which may practically depend upon the timing of cART initiation and the sensitivity of the virologic assays employed, in the present study, we treated SIV-infected rhesus macaques with cART starting 65 days post-infection to better match the conditions of the Lim et al. study. Animals were then treated with six oral doses of the TLR7 agonist VES once every 2 weeks beginning 17–19 weeks after cART initiation when the population of residual infected cells still feature a degree of residual virologic expression activity. Innate and adaptive immunomodulatory effects and virologic effects were monitored in blood plasma, PBMC, and tissues, including the evaluation of blood specimens collected at both 24 and 48 h after each VES administration. Plasma viral loads were monitored using a highly sensitive quantitative reverse transcription polymerase chain reaction (qRT-PCR) assay with a threshold sensitivity of 15 vRNA copies/ml (40). We additionally utilized surgically implanted catheters placed in the hepatic portal vein with venous access ports in a subset of study animals to assess with maximum sensitivity potential viral induction from gut tissue sources following oral administration of VES that might go undetected in peripheral venous blood after first pass clearance through the liver.

## MATERIALS AND METHODS

### Animals and treatments

Ten purpose-bred, Indian-origin rhesus macaques (*Macaca mulatta*, aged 4.6–8.0 years at study initiation, 6 females and 4 males) were housed at NIH-Bethesda. Prior to study initiation, all animals were free of cercopithecine herpesvirus 1, SIV, simian type-D retrovirus, and simian T lymphotropic virus type 1. Animals were screened for the Mamu major histocompatibility complex (MHC) Class I alleles Mamu-A*01, -B*08, and -B*17 using allele-specific PCR (41). All 10 animals were negative for the Mamu-B*08 and Mamu-B*17 MHC alleles. Six animals were positive for the Mamu-A*01 MHC allele (Table S1). Animals were each intravenously inoculated with 10^4^ infectious units (titer determined on TZM-bl cells, as previously described [42]) of the barcoded virus SIVmac239M (43). At 65 dpi, all 10 animals started on a daily, subcutaneously-injectable cART regimen consisting of tenofovir disoproxil fumarate (TDF), emtricitabine (FTC), and dolutegravir (DTG), coformulated and administered as previously described (44, 45). Apart from animal 12M153, which was euthanized for clinical reasons unrelated to the study 40 weeks after cART initiation while still receiving cART, all study animals received continuous daily cART for at least 54 weeks. Vesatolimod or vehicle control (0.005% propyl gallate in water) administrations were performed via oral gavage to sedated animals once every 2 weeks for six total administrations per animal beginning 15–17 weeks after cART initiation. Vesatolimod was administered at 0.15 mg/kg in a volume of 1 mL/kg. Drug vehicle was administered to the control group animals at 1 mL/kg. Daily cART was discontinued 28–29 weeks after the final VES administration for the VES group animals except for 12M153. To conserve animal resources, control group animals were maintained on cART and repurposed for another study not described here. Hepatic portal vein catheters to enable collection of hepatic portal vein blood were surgically placed in five study animals (three VES group animals; two vehicle control group animals) based on a modified version of a previously reported procedure (46). For the placement of hepatic portal vein catheters, each animal was sedated and the spleen was gently externalized from the abdominal cavity through an oblique subcostal incision. A mid-branch of the splenic vein was located, isolated from surrounding tissue, and then partially transected with suture ligatures placed proximal and distal to the incision to control bleeding. A polyurethane catheter was advanced through the splenic vein toward the portal vein using a J guide wire. The catheter was then secured with the proximal suture looping the vessel. The guide wire was removed, and the catheter was tested and then flushed with physiological saline. The spleen was reintroduced into the abdomen and a small conventional port pocket was placed proximal to the original incision. The intravenous catheter was then trimmed and attached to a standard vascular access port. The port was flushed with saline and catheter lock solution (alteplase, Genentech). The abdomen and incision were then closed with absorbable suture using standard techniques. All work involving research animals was conducted under a protocol (AVP-056) approved by the Animal Care and Use Committee of the National Cancer Institute, National Institutes of Health (NIH) in NIH-Bethesda facilities. NIH-Bethesda is accredited by AAALAC International and follows the Public Health Service Policy for the Care and Use of Laboratory Animals (Animal Welfare Assurance Number D16-00602). Animal care adhered to the standards outlined in the Guide for the Care and Use of Laboratory Animals (National Research Council; 2011; National Academies Press; Washington, D.C.) in accordance with the Animal Welfare Act.

### Sample collection and preparation

Whole blood, excisional lymph node biopsies, and rectal pinch biopsies were collected from sedated animals. Whole blood was collected in EDTA Vacutainer Tubes (BD). During the VES treatment phase of the study, peripheral venous blood was collected immediately prior to and at 24 h, 48 h, and 7 days following each VES or vehicle control dose. For animals with hepatic portal vein catheters, hepatic portal venous blood was collected once per week for 10 consecutive weeks, beginning 3 weeks prior to the first VES/vehicle dose and generally at 24 h post-VES/vehicle dose during the VES treatment phase of the study. For sampling from the portal vein, the area over the venous access port was clipped and prepped, and the port was accessed with a Huber needle attached to a 3 mL syringe. An initial 3 mL of blood was removed from the port and discarded, followed by collection of a blood sample to be used for analysis. The port then was flushed with sterile saline and then alteplase (Genentech). Plasma for vRNA quantification was separated from whole blood, further clarified by sequential centrifugation, and then stored at –80°C. Following plasma separation, the blood cellular component was resuspended in an equivalent volume of PBS and then PBMCs were isolated by Ficoll-Paque Plus (GE Healthcare) gradient centrifugation. Portions of isolated PBMC samples were cryopreserved as dry cell pellets for CA-vRNA and CA-vDNA quantification. Rectal pinch biopsies were obtained using biopsy forceps by direct visualization. Freshly collected LN and rectal tissue specimens were collected into tubes containing zirconium microbeads (for later tissue dissociation) and snap-frozen in liquid nitrogen or into tubes containing 4% paraformaldehyde.

### Plasma viral loads

SIV RNA in plasma derived from peripheral venous blood or from hepatic portal venous blood was quantified using a quantitative reverse-transcription PCR (qRT-PCR) assay with a threshold quantification limit of 15 vRNA copies/mL or 24 vRNA copies/mL, respectively, as previously described (47). The higher threshold quantification limit for plasma derived from hepatic portal venous blood was due to collection of smaller blood volumes and smaller analyzed plasma volumes. Plasma viral loads in samples for which vRNA levels were below threshold (i.e., vRNA was not detected) are reported as “less than 15 vRNA copies/mL” or “less than 24 copies/mL.”

### Cell-associated viral loads

Cell-associated vRNA and vDNA levels in PBMC and biopsy tissues were analyzed essentially as described previously, using qPCR and qRT-PCR assays targeting a conserved sequence in SIVmac239 *gag* (48, 49). Threshold sensitivity for this assay is dependent on the amount of input sample available for testing.

### Interferon-stimulated gene expression

Relative interferon-stimulated gene (ISG) expression levels were determined by a qRT-PCR-based expression array, essentially as previously described (34). Briefly, cell-associated RNA was extracted from thawed, previously cryopreserved PBMC pellets using the RNeasy Plus Mini Kit (Qiagen) according to the manufacturer’s instructions, quantified by NanoDrop (ThermoFisher), and quality-checked by bioanalyzer using the RNA 6000 Nano kit (Agilent) according to the manufacturer’s instructions. Extracted RNA was used to generate cDNA using the RT^2^ First Strand Kit, with selected ISG sequences in the cDNA quantified by qPCR on a QuantStudio 5 real-time PCR system (ThermoFisher) using a customized version of the Rhesus Macaque Type I Interferon Response RT² Profiler PCR Array containing only the *IFI27*, *ISG15*, and *Mx1* genes with the RT² SYBR Green ROX qPCR Mastermix (Qiagen), all according to the manufacturers’ instructions. Fold regulation was calculated by the ΔΔCT method using the RT^2^ Profiler PCR Array Data Analysis Webportal (https://www.qiagen.com/us/shop/genes-and-pathways/data-analysis-center-overview-page/).

### Flow cytometric assays

Antibodies and reagents were obtained from BioLegend unless indicated otherwise, and data analysis was performed using FCS Express (*De Novo* Software). All antibodies were titrated to determine optimal test amount, using identical experimental conditions and cytometer configurations. Panel validation and population gating were performed using relevant biological and fluorescence-minus-one (FMO) controls. Leukocyte immunophenotyping was performed with the following surface staining panel: CD4 BUV395 (L200, BD Biosciences), CD123 BV510 (6H6), CD27 BV605 (O323), CD95 BV711 (DX2), CD16 BV785 (3G8), CD20 PerCP-Cy5.5 (2H7), CD14 APC (M5E2), HLA-DR Alexa Fluor 700 (L243), CD3 APC-Cy7 (SP34-2, BD Biosciences), CD38 PE (OKT10, NIH Nonhuman Primate Reagent Resource), CD28 ECD (CD28.2, Beckman Coulter), CD56 PE-Cy5 (B159, BD Biosciences), and CD8α PE-Cy7 (SK1). Briefly, 100 µL EDTA-anti-coagulated whole blood was incubated for 20 min at room temperature, in the dark (RTD) with the surface staining panel. Samples were then treated for 10 min at RTD with 1× FACS Lyse buffer (BD Biosciences), washed with PBA (PBS, 0.5% bovine serum albumin, 0.05% sodium azide), and then treated for 20 min at RTD with Cytofix/Cytoperm buffer (BD Biosciences). Samples were washed with 1× Perm Wash (BD Biosciences) and incubated for 30 min at 4°C with an intracellular staining panel containing Ki67 FITC (B56, BD Biosciences) and CD69 PE-Cy7 (TP1.55.3, Beckman Coulter) antibodies. Samples were washed again with 1× Perm Wash and resuspended in PBA, and approximately 200,000 CD3+ T cells were acquired for each sample using a Fortessa X-20 flow cytometer (BD Biosciences).

### Tissue staining

ISG15 (rabbit anti-ISG15, HPA004627; Sigma-Aldrich) and MxA (mouse anti-MxA, MABF938; Millipore Sigma) immunohistochemical staining was performed on formalin-fixed, paraffin-embedded LN, duodenal, and rectal biopsy tissue sections of 5 µm as previously described (50). All slides were scanned at high magnification (×200) using the ScanScope AT2 System (Aperio Technologies). Whole-tissue scans, with 2 sections per slide, were segmented into small regions of interest to allow easier data extraction, but two entire tissue sections were analyzed per time point and animal. The percentage area of ISG15- or MxA-positive staining was quantified using Cell Profiler and normalized to tissue area.

### Statistical methods

Statistical analysis was done using custom R scripts (R Core Team, 2021. R: A language and environment for statistical computing. R Foundation for Statistical Computing, Vienna, Austria. URL https://www.R-project.org/). Where within-group distributions were generally normally distributed (as determined by Shapiro-Wilk test *P*-values > 0.05), *t*-tests were used for group comparisons. Otherwise, the non-parametric Wilcoxon rank sum test was used for comparisons. Unless otherwise noted, all comparisons were unpaired and two-sided. For ISG expression analyses and analyses of flow cytometric assessment of NK cell and monocyte marker expression, within-animal repeated measurements showed clear animal trends and no clear changes with additional doses. To avoid pseudoreplication, within animal, repeated measurements were averaged and compared using unpaired two-sided Wilcoxon rank sum tests (within group values were not normally distributed). For PBMC and tissue RNA:DNA ratio analyses, RNA:DNA ratio values were log_2_ transformed. The transformed data were generally normally distributed and pre/post time points were compared using unpaired two-tailed *t*-tests. To account for the multiple comparisons across the different genes within each time point, a Bonferroni correction was applied to the resulting *P*-values. For T cell activation analyses, group distributions often deviated from normality (Shapiro-Wilk test *P*-values < 0.05) and were, therefore, compared using unpaired two-sided Wilcoxon rank sum tests. To account for the multiple comparisons across the different T-cell types, a Bonferroni correction was applied to the resulting *P*-values. To determine if PVLs at 24 h or 48 h were higher than the pre-dose time point across all doses for the VES group or control animals, a one-sided matched non-parametric (Wilcoxon Signed-Rank Test) comparison was performed. For this analysis, all below-threshold (<15 vRNA copies/mL) values were set at a value of 14 copies/mL. To compare patterns of PVL changes at 24 h or 48 h relative to pre-dose time points between the study groups, each post-dose PVL measurement for each study animal was categorized as increasing relative to pre-dose, decreasing relative to pre-dose, or unchanged. Two-sided Fisher’s exact tests were used to compare the resulting counts for each group at each time point. For analysis of vDNA changes, vDNA values were positively skewed. Natural log transformation brought within-group values closer to normally distributed, allowing the use of paired *t*-tests.

## RESULTS

### Study design and virologic suppression on cART

**Fig 1:**
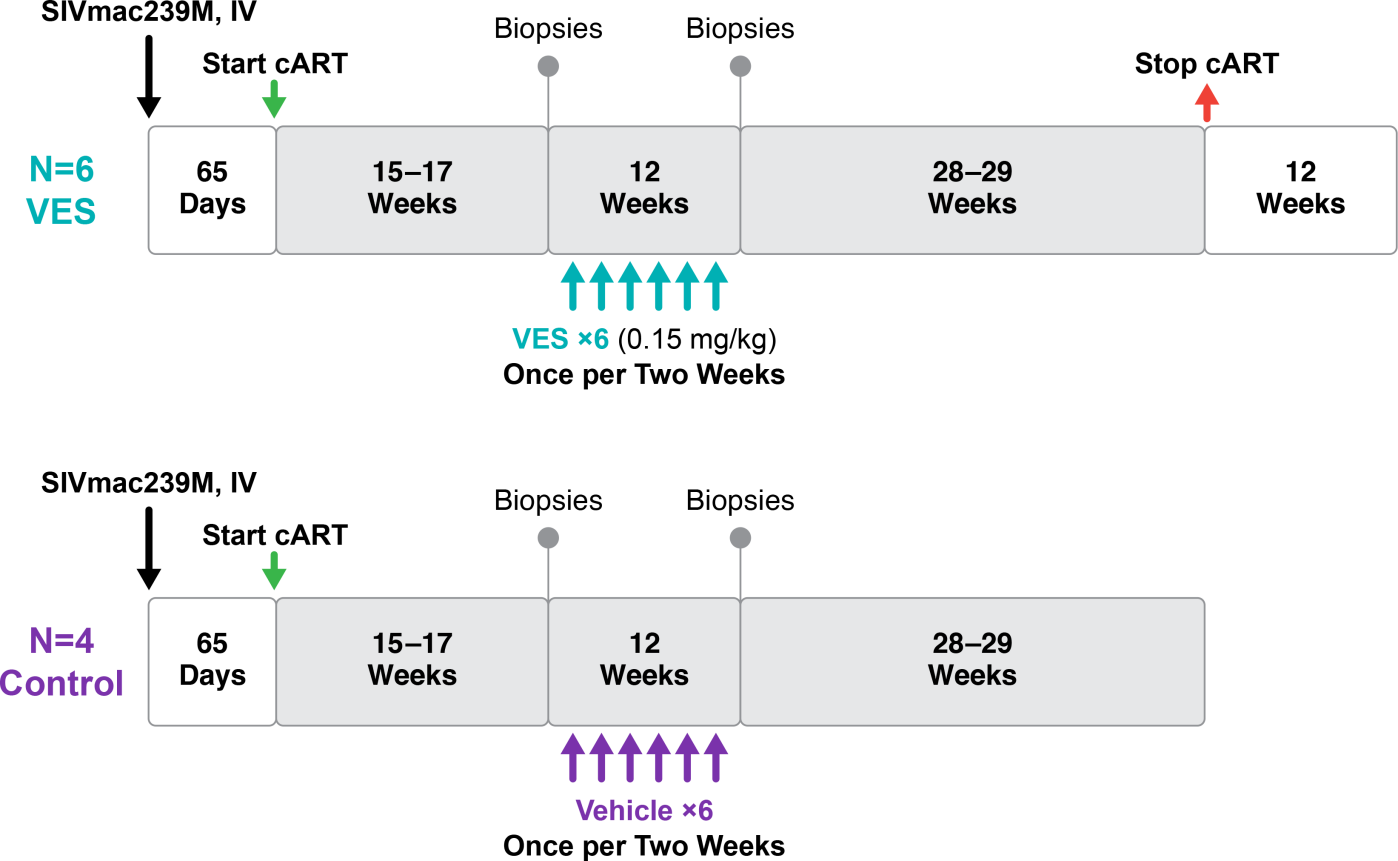
Study design. Shown is the study design schema depicting the timing of SIVmac239M infection, cART initiation, VES or vehicle control administrations, biopsy collections, and cART cessation.

**Fig 2:**
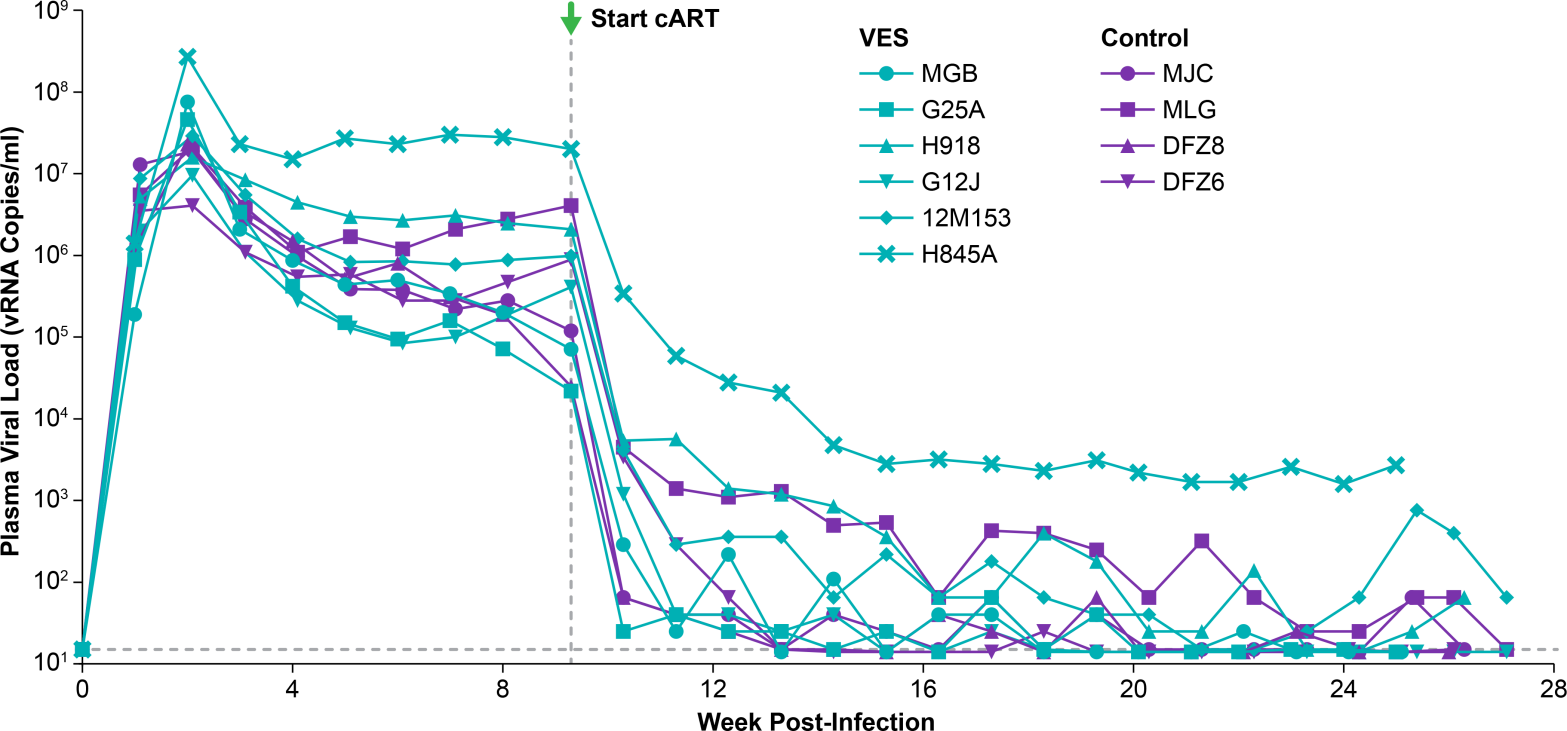
Plasma viral loads prior to VES treatment. Longitudinal quantification of vRNA in plasma for each study animal is shown for the first 26–28 weeks of the study, encompassing time points prior to cART initiation through the first 17–19 weeks of cART. Animals assigned to the VES treatment group are shown with teal plot symbols, while those assigned to the vehicle control group are shown with purple plot symbols. The horizontal dashed line represents the threshold sensitivity for the PVL assay utilized (15 vRNA copies/mL).

 Ten Indian-origin rhesus macaques (RMs) were intravenously infected with SIVmac239M (43) and treated with a daily three-drug cART regimen (44, 45) starting at 65 days post-infection (dpi) (Fig. 1). Prior to cART initiation, PVLs ranged from 2.2 × 10^4^ to 2.0 × 10^7^ vRNA copies/mL, with one animal (H845A) displaying notably higher pretreatment PVLs than the other study animals (Fig. 2). In animals with pretreatment PVLs < 10^6^ vRNA copies/mL, PVLs declined to 15 copies/mL or lower within 4–12 weeks of cART initiation, while it took 14–18 weeks of cART for PVLs to decline to 15 copies/mL or lower in those with pretreatment PVLs between 10^6^ and 10^7^ vRNA copies/mL (Fig. 2). In animal H845A, which had a pretreatment PVL > 10^7^ vRNA copies/mL, PVLs declined by ~4 logs within the first 5 weeks of cART but remained above 10^3^ vRNA copies/mL through 17 weeks on cART. Apart from one VES group animal (12M153) that was euthanized for veterinary reasons unrelated to the study 11 weeks after the final VES dose, each SIV-infected RM received daily cART for at least 56 weeks. Study animals were divided into a VES treatment group (*N* = 6; median pre-cART PVL = 7.0 × 10^5^ vRNA copies/mL, range 2.2 × 10^4^–2.0 × 10^7^ vRNA copies/mL) and a vehicle control group (*N* = 4; median pre-cART PVL = 5.0 × 10^5^ vRNA copies/mL, range 2.5 × 10^4^–4.1 × 10^6^ vRNA copies/mL) (Fig. 1), balanced for pre-cART PVLs (Fig. S1 and Table S1). Beginning 17–19 weeks after cART initiation, VES treatment group animals then received six oral doses of VES once every 2 weeks, while vehicle control group animals received drug vehicle according to the same schedule (Fig. 1).

### Innate immunomodulation following VES treatment

**Fig 3:**
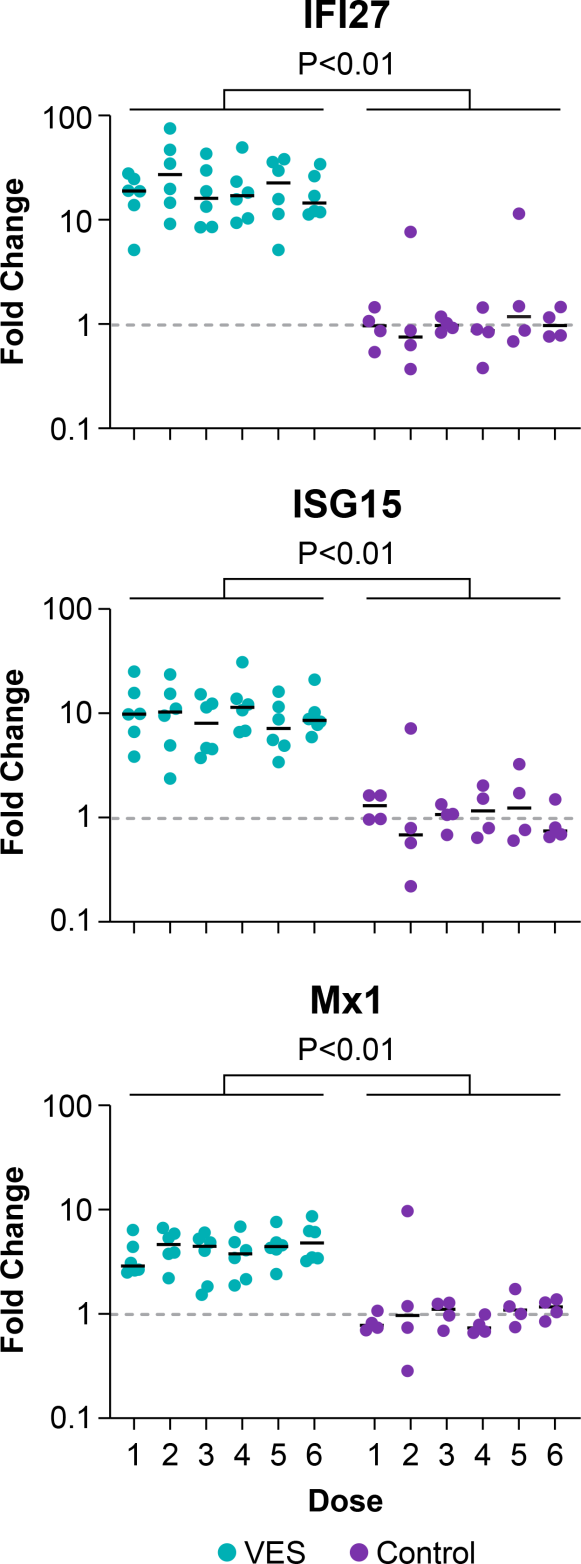
Increased IFN-stimulated gene expression following VES treatment. RNA expression was quantified for the IFN-stimulated genes *IFI27*, *ISG15*, and *Mx1* in PBMCs collected immediately prior to and at 24 h following each dose of VES or vehicle. Shown is the fold change in RNA expression for each animal, gene, and dose in PBMCs collected at the 24 h time point compared with PBMCs collected prior to the corresponding VES/vehicle dose for VES (teal) and vehicle control (purple) animals. Values > 1 indicate upregulated RNA expression, while values < 1 indicate downregulated RNA expression. The expression of all three ISGs was significantly upregulated in VES-treated animals compared with controls (unpaired two-sided Wilcoxon rank sum tests).

 We have previously shown that stimulation of TLR7 following VES administration to RMs results in the increased expression of specific interferon-stimulated genes (ISGs) (34), likely due to increased type I interferon (IFN) production by plasmacytoid dendritic cells (pDCs), which express TLR7 (51). In our prior work, we found that numerous ISGs were significantly upregulated following oral VES dosing, with *IFI27*, *ISG15*, and *Mx1* among those genes that showed the most dramatic degree of upregulation (34). To confirm the administration of immunomodulatory doses of VES in the present study, we first evaluated the RNA expression level for each of these three ISGs in PBMCs immediately prior to and at 24 h after each VES or vehicle control administration (Fig. 3). The expression levels of each of these three ISGs were significantly upregulated at 24 h in VES-treated animals compared with vehicle controls, with mean expression increases of 22.4-fold, 10.6-fold, and 4.3-fold for *IFI27*, *ISG15*, and *Mx1*, respectively (Fig. 3). The ISG expression responses were relatively consistent across VES doses, with no evidence of increasing or decreasing responsiveness with successive VES administrations.

**Fig 4:**
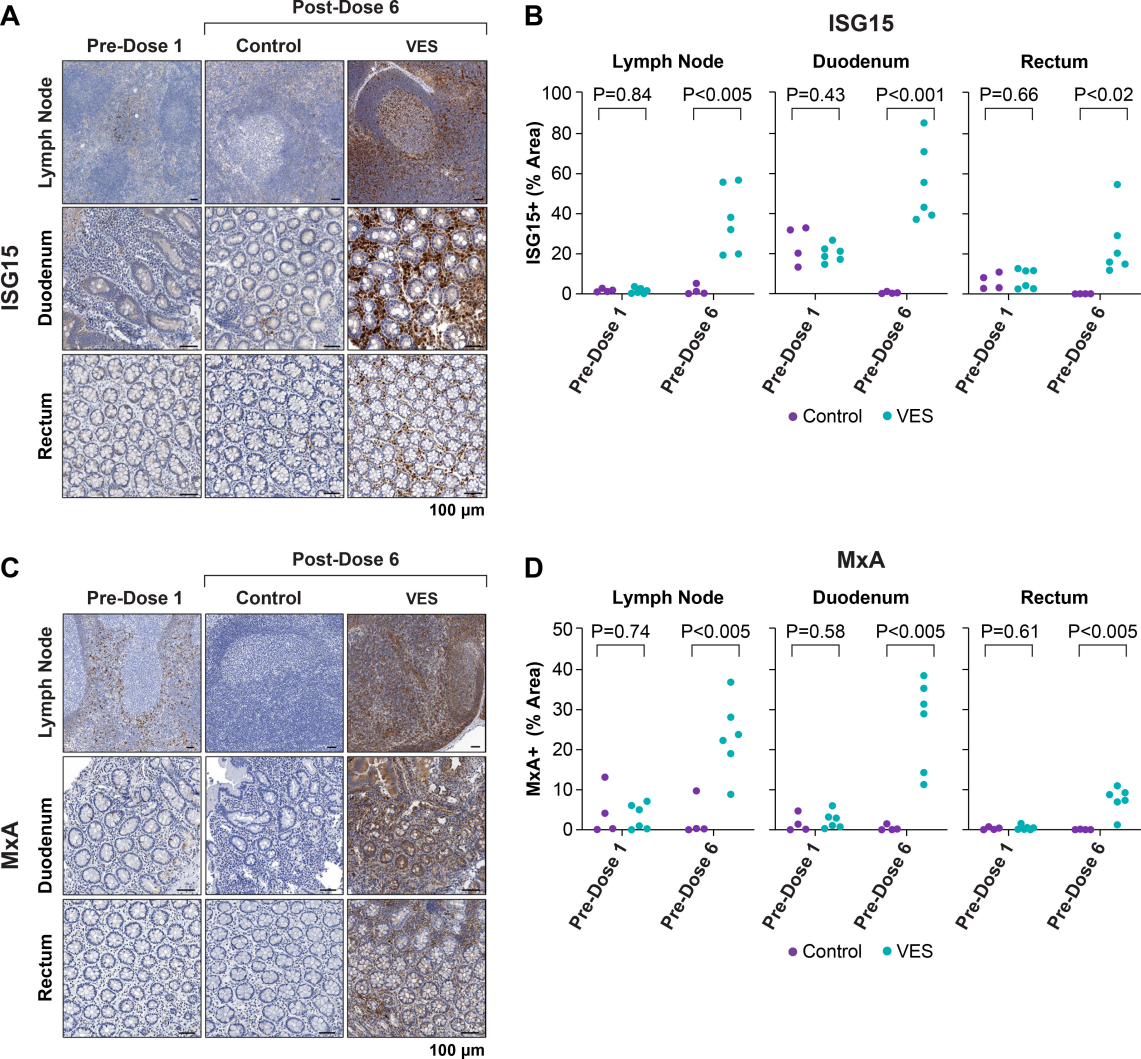
Increased expression of ISG15 and MxA protein in tissues following VES treatment. Representative images of ISG15 (**A**) and MxA (**C**) protein staining (brown) in LN, duodenal, and rectal biopsy tissues collected prior to the first dose of VES/vehicle (Pre-dose 1) and 24 h after vehicle (control) or VES dose 6 (Post-dose 6) are shown. Scale bars: 100 µm. Quantitative image analysis of ISG15 (**B**) and MxA (**D**) staining was performed for each tissue type for all 10 study animals prior to dose 1 (Pre-dose 1) and following dose 6 (Post-dose 6). There was no statistical difference between the groups for either protein in any analyzed tissue prior to the first dose, but at 24 h after VES/vehicle dose 6, staining for both ISG15 and MxA was significantly higher in VES-treated animals than in controls in all three analyzed tissues (two-sided *t*-tests).

 To determine if oral VES administration also induced innate immunomodulation in key tissue sites of SIV replication and persistence on cART, we quantified ISG15 (Fig. 4A and B) and MxA (Fig. 4C and D) protein levels by immunohistochemistry in peripheral lymph node, duodenal, and rectal biopsy tissues collected from VES and vehicle control group animals prior to VES dose 1 and 24 h after VES dose 6. Prior to VES administration, there were no significant differences between VES and vehicle control animals for either ISG15 or MxA staining in any evaluated tissue (Fig. 4B and D). However, following VES dose 6, both ISG15 and MxA staining were significantly higher in VES treated animals compared with vehicle controls in all three tissues (Fig. 4B and D). The largest magnitude differences between VES and vehicle control animals for both ISG15 and MxA staining were measured in upper gastrointestinal tissue (duodenum), while the smallest differences between VES and vehicle control animals were measured in lower gastrointestinal tissue (rectum) (Fig. 4B and D).

### Leukocyte responsiveness to VES treatment

**Fig 5:**
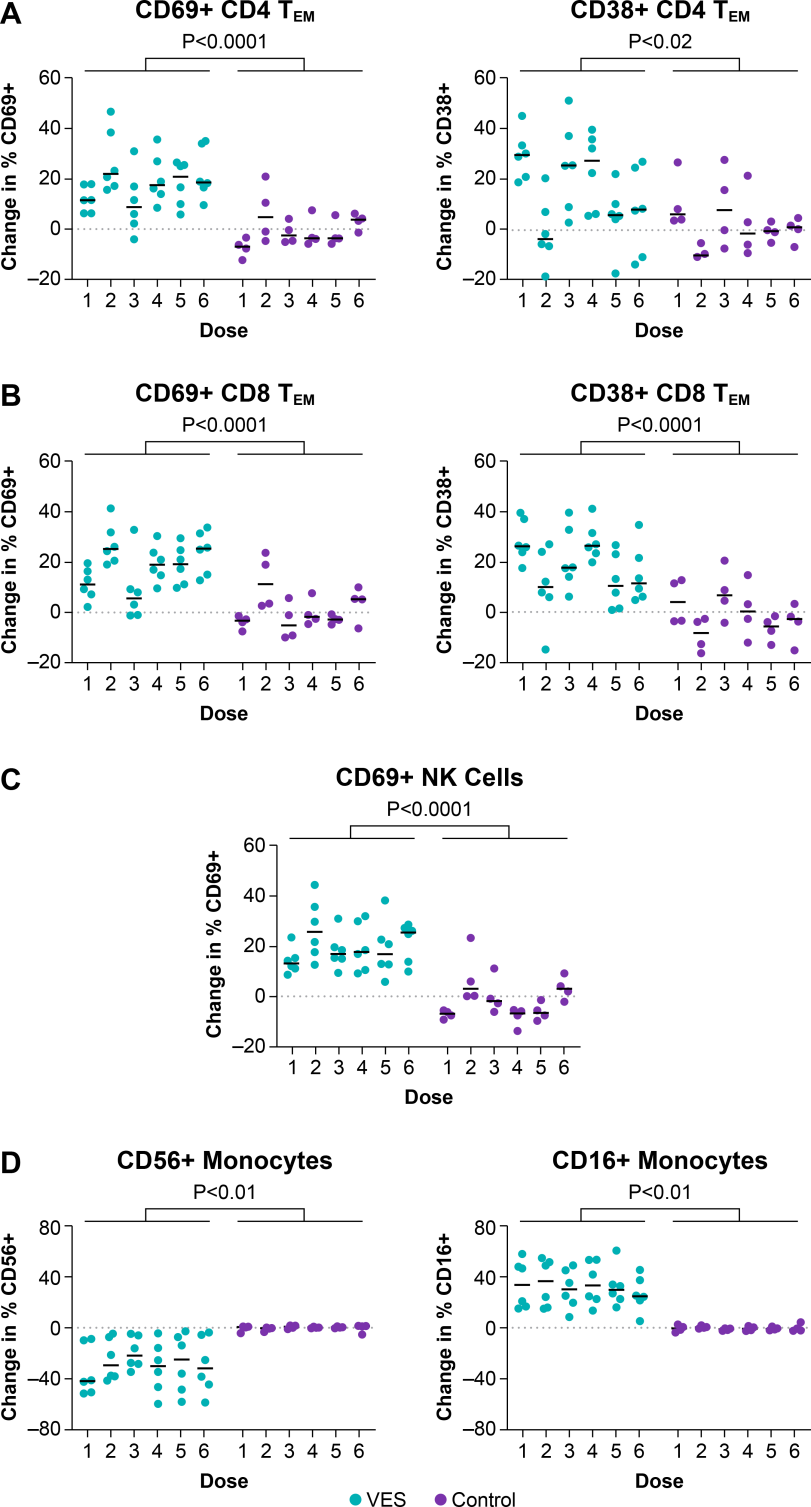
Response of immune cell populations in blood to VES treatment. Immune cell populations in blood were analyzed longitudinally by flow cytometry. Shown is the raw difference in the percentage of each indicated blood immune cell population expressing the indicated marker at 48 (**A–C**) or 24 (**D**) h after each VES/vehicle dose compared with blood cells collected immediately prior to the corresponding VES/vehicle dose for each animal (% of cells expressing marker following dose minus % of cells expressing marker prior to dose). Following VES/vehicle administration, there were significant increases in the percentage of CD4+ (**A**) and CD8+ T_EM_ cells (**B**) expressing CD69 and CD38, the percentage of NK cells (**C**) expressing CD69, and the percentage of monocytes (**D**) expressing CD16, as well as a significant decrease in the percentage of monocytes expressing CD56 in VES-treated animals compared with controls (unpaired Wilcoxon rank sum tests).

 We and others have previously reported that TLR7 stimulation in SIV/SHIV-infected RMs on cART leads to characteristic phenotypic changes in CD4+ T cells, CD8+ T cells, NK cells, and monocyte populations in blood (31–36, 38, 39, 52–54). We utilized longitudinal flow cytometry performed on blood samples collected prior to and following each VES or vehicle control dose to analyze VES-induced phenotypic changes in each of these leukocyte populations. For each animal, we calculated the change in marker expression by subtracting the raw percentage of cells expressing each assessed marker prior to each VES/vehicle dose from the raw percentage of cells expressing the same marker after the corresponding VES/vehicle dose (% of cells expressing marker following dose minus % of cells expressing marker prior to dose). We first evaluated the expression of the activation markers CD69 and CD38 on CD4+ T cell memory subsets, as activation of CD4+ T cells harboring viral genomes is a proposed mechanism by which TLR7 stimulation may induce virus expression. VES-induced activation of CD4+ T cells was primarily displayed by CD4+ T effector memory cells (CD95+CD28−; T_EM_). At 48 h post-VES administration, CD69 expression was significantly elevated on CD4+ T_EM_ cells in the VES-treated animals compared with controls (mean change in % CD69+: +18 for VES group, no change for vehicle controls) (Fig. 5A), but there were no significant differences in CD69 expression between VES-treated animals and controls on CD4+ T central memory (CD95+CD28+; T_CM_) or T naïve (CD95−CD28+; T_N_) subsets (Fig. S2A). CD38 expression was also significantly elevated on CD4+ T_EM_ cells 48 h post-dose in the VES treated animals compared with controls (mean change in % CD38+:+15 for VES group vs +1.6 for vehicle controls) (Fig. 5A). Unlike CD69, CD38 expression also significantly increased on CD4+ T_CM_ in VES-treated animals compared with vehicle controls though the magnitude of the effect was smaller than for CD4+ T_EM_ cells (mean change in % CD38+: +3.2 for VES group vs −1.3 for vehicle controls) (Fig. S2B). VES treatment did not lead to a measurable change in CD38 expression on CD4+ T_N_ cells (Fig. S2B).

 CD8+ T cells were broadly more responsive to VES treatment than CD4+ T cells, with significant activation marker increases for all 3 analyzed CD8+ T cell memory subsets (T_EM_, T_CM_, T_N_). At 48 h post-VES administration, the percentage of CD8+ T cells expressing CD69 and CD38 increased significantly in VES-treated animals compared with vehicle controls for CD8+ T_EM_, T_CM_, and T_N_ memory subsets (Fig. 5B ; Fig. S2C and D). Like CD4+ T cells, of the CD8+ memory subsets analyzed, T_EM_ cells showed the greatest degree of activation in response to VES treatment (mean change in % CD69+ and % CD38+: +18 and +19, respectively) (Fig. 5B). Increases in activation marker expression on CD8+ T_CM_ and T_N_ cells were also significant but less robust (mean change in % CD69+ and % CD38 T_CM_: +3.4 and +6.5, respectively; mean change in % CD69+ and % CD38 T_N_: +11 and +4.6, respectively) (Fig. S2C and D).

 In addition to T cell activation, modulation of innate immune cell populations following *in vivo* administration of TLR7 agonists has also been previously reported (33–36, 38, 39, 53, 54). Consistent with these prior studies, we observed significant activation of NK cells in blood in our VES-treated animals. At 48 h post-dose, the percentage frequency of CD69+ NK cells in blood increased by 20 percentage points relative to pre-treatment in VES-treated animals, compared with a 1.7 percentage point decline in CD69+ NK cells in vehicle control animals (Fig. 5C). In addition, we previously showed that blood monocytes, which express TLR7 and are, therefore, potentially directly impacted by agents that stimulate TLR7, displayed dramatic phenotypic changes in response to VES administration (34). Although in humans only a very small fraction of monocytes express CD56 (55), in healthy rhesus macaques, cell-surface CD56 is typically expressed by >80% of monocytes in blood (56). In the present study, the mean percentage of circulating monocytes expressing CD56 significantly declined from 91% to 64% at 24 h post-dose in VES-treated animals, while the percentage of monocytes expressing CD56 did not change in control group animals (Fig. 5D). Conversely, and consistent with our prior work (34), the frequency of CD16 expression on monocytes significantly increased following VES treatment. The mean percentage of CD16+ monocytes increased from 12% prior to VES administration to 44% at 24 h post-VES administration, while in vehicle controls, CD16+ monocytes declined slightly from 6% to 5% during the same time interval (Fig. 5D).

### Plasma viral loads during VES administration

**Fig 6:**
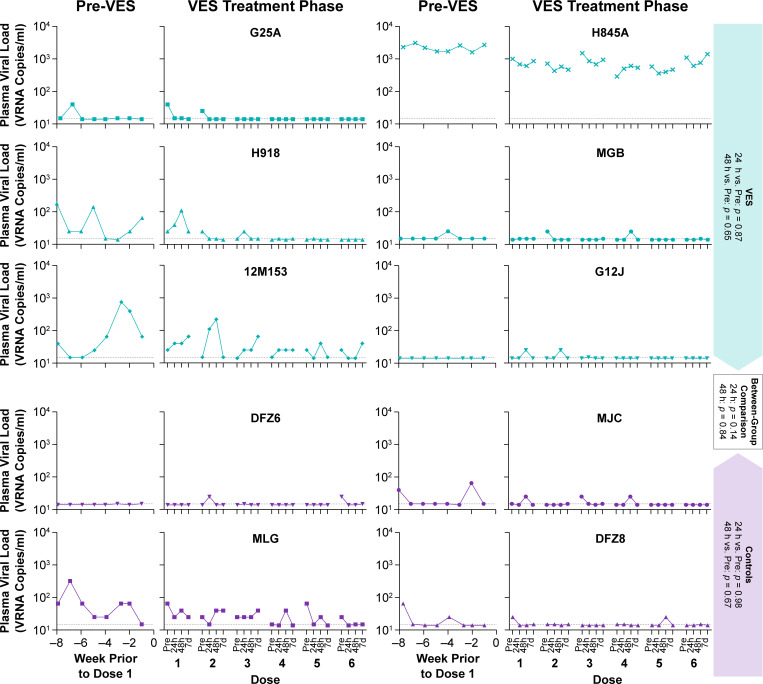
No evidence of plasma viral load increases following repeated VES treatment**.** SIV RNA was quantified in plasma immediately prior to and at 24 h, 48 h, and 7 days after each VES or vehicle control administration. Shown for each animal are the longitudinal SIV RNA measurements for 8 weeks prior to the first VES/vehicle control dose (Pre VES) and for the pre- and post-dose time points for each VES/vehicle administration. The threshold sensitivity for the PVL assay utilized (15 vRNA copies/ml) is represented on each plot by a horizontal dashed line. Across animals and doses, PVLs were not significantly higher at 24 h or at 48 h compared with the “pre” time points for either the VES or vehicle control groups (one-sided Wilcoxon signed-rank test). There were also no significant differences between the VES and control group animals in the number of doses for which PVLs measured at either 24 h or 48 h were higher than the “pre” time point (Fisher’s exact test).

 Lim et al. reported that in SIV-infected macaques on cART, repeated administration of the TLR7 agonist VES or the related compound GS-986 induced transient >10-fold PVL increases that peaked 24–48 h after each TLR7 agonist dose. In the Lim study, plasma vRNA increases were not observed following the first 2–3 doses of TLR7 agonist but were detected following subsequent doses, suggestive of an initial “priming” phase that preceded robust viral reactivation. We, therefore, monitored PVL in our VES and vehicle control group animals immediately prior to and at 24 h, 48 h, and 7 days after each VES or vehicle administration (Fig. 6). Across all doses, PVLs were not significantly higher than the corresponding pre-dose levels at either 24 h or 48 h for either VES-treated or control animals, suggesting that the measured PVL fluctuations represent background biological variability, rather than VES-mediated virus induction. In total across all animals, 36 doses of VES and 24 doses of vehicle were administered. At 24 h in the VES treatment group, PVLs were higher than pre-dose levels following 11 total doses, but were lower than pre-dose levels following 11 total doses, and were unchanged following 14 total doses (*P* = 0.87). Similarly, at 24 h in the vehicle control group, PVLs were higher than pre-dose levels following 2 total doses, lower than pre-dose levels following 9 total doses, and unchanged following 13 total doses (*P* = 0.98). At 48 h in the VES treatment group, PVLs were higher than pre-dose levels following 12 total doses, but were lower than pre-dose levels following 10 total doses, and were unchanged following 14 total doses (*P* = 0.65). Again, the patterns were similar for the vehicle control group at 48 h, with PVLs higher than pre-dose levels following 6 total doses, lower than pre-dose following 8 total doses, and unchanged following 10 total doses (*P* = 0.67) (Fig. 6). Given the observation in the Lim study that PVL induction was not observed following the first 2–3 doses of TLR7 agonist, we repeated this analysis focusing only on the final three doses of VES (doses 4–6). We again observed no significant differences between pre-dose PVLs and PVLs at 24 h or 48 h for VES treated animals (24 h: *P* = 0.82; 48 h: *P* = 0.5) or for vehicle control animals (24 h: *P =* 0.98; 48 h: *P* = 0.5) (Fig. 6).

 In addition to these within-group comparisons, we also asked whether there were any significant differences between the VES and vehicle control group animals in the number of doses for which plasma viral loads at 24 h, 48 h, or either time point were higher than the corresponding pre-dose time points. We again found no evidence of VES-induced increases in PVL. There were no significant differences between the VES and vehicle control groups in PVL trends at 24 h (*P* = 0.14), 48 h (*P* = 0.84), or when examining PVL increases occurring at either 24 h or 48 h (*P* = 0.43) (Fig. 6).

 Because VES is administered orally and is cleared with first-pass hepatic metabolism, we explored the possibility that VES-mediated virus induction might be most robust in residual infected cells residing within intestinal tissues. We further reasoned that virus produced from gut-resident cells may not be readily detectable in peripheral blood because blood from the gastrointestinal tract is transported to the liver for filtration via the hepatic portal vein prior to reaching the systemic circulation. To quantify hepatic portal vein PVLs (i.e., blood from the intestine that has not yet been filtered by the liver), we surgically placed intravenous catheters with access ports in five study animals (three VES group, two vehicle control group). We collected blood from these portal vein catheters weekly for 3 weeks prior to the first dose of VES or drug vehicle, and at 24 h or 48 h after each VES or vehicle dose. Portal vein PVLs following VES or vehicle control administrations remained at or below pre-dose levels for all 5 animals, with no measurable increases associated with VES administration (Fig. S3A). Apart from animal H845A, which had the highest pre-cART PVLs and maintained quantifiable PVLs during cART, the portal vein PVLs for each of the remaining two VES group animals remained at or below the PVL assay quantification limit for portal vein plasma (24 vRNA copies/mL) throughout the VES treatment phase of the study. There was good agreement between portal vein PVLs and peripheral blood PVLs collected from the same animals at the same time points (Rs = 0.82, Spearman rank correlation), with no apparent trend for higher PVLs in portal vein blood following VES administrations (Fig. S3B).

### Cell- and tissue-associated viral loads during VES administration

**Fig 7:**
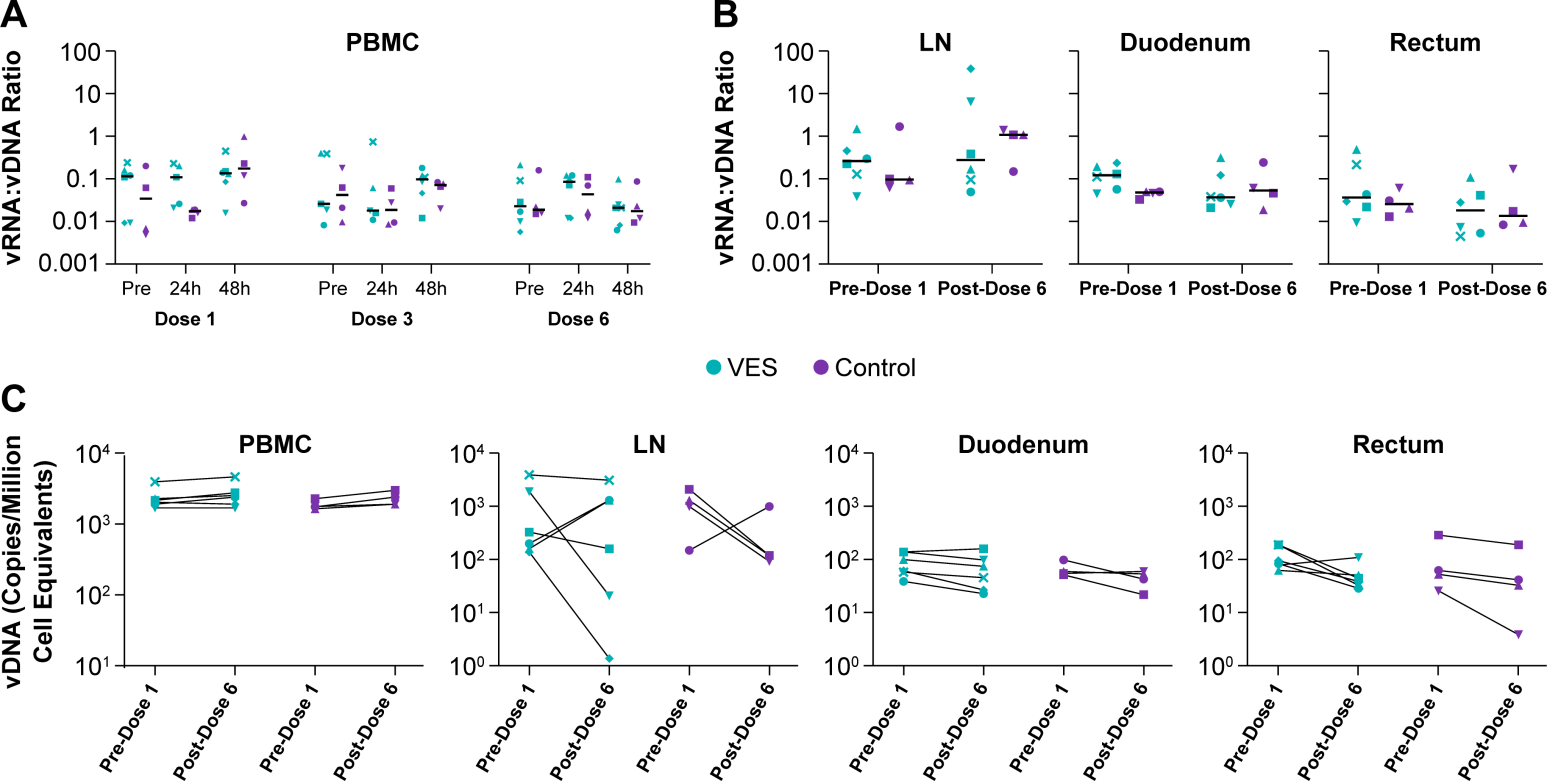
No evidence of increased viral RNA expression or decreased viral DNA levels associated with repeated VES treatment. (**A, B**) Cell/tissue-associated vRNA and vDNA content were quantified within the same samples and the ratio of these values calculated. The calculated ratio in PBMC samples collected immediately prior to (pre) and at 24 h and 48 h following VES/vehicle doses 1, 3, and 6 are shown in **A**. The calculated ratio in LN, duodenal, and rectal tissue specimens collected and snap frozen prior to the first VES/vehicle dose (pre) and at 24 h following dose 6 are shown in **B**. There were no significant differences between the VES and control group animals in the vRNA:vDNA ratio for any of the analyzed time points or samples (unpaired two-sided *t*-tests). (**C**) Viral DNA quantification was performed for PBMC, LN, duodenum, and rectum specimens collected prior VES/vehicle dose 1 and 24 h after VES/vehicle dose 6. Shown are the changes in vDNA content in each sample type for each study animal. There were no significant differences in vDNA levels between pre-dose 1 and post-dose 6 samples in any analyzed specimen type for either treatment group (paired *t*-tests).

 To determine if VES-mediated TLR7 stimulation led to measurable increases in cell-associated unspliced vRNA expression from persistent vDNA genomes, we quantified both vRNA and vDNA in the same PBMC and tissue specimens and then calculated the vRNA-to-vDNA ratio within each sample. This approach provides a “transcriptional ratio” for each sample or the vRNA copy number for each vDNA genome present within the same sample. We first evaluated the vRNA:vDNA ratio in PBMC specimens collected prior to and at 24 and 48 h after VES or vehicle doses 1, 3, and 6 (Fig. 7A). There were no significant differences between VES-treated and vehicle control animals at any of the analyzed time points, with no consistent increases in vRNA levels at any of the post-treatment time points compared with the corresponding pretreatment time point (Fig. 7A). Cell-associated vRNA levels in PBMC remained <1 vRNA copy per vDNA copy across all study animals and time points (median: 0.03, range: 0.005–0.7 vRNA copies/vDNA copy) (Fig. 7A).

We next compared the vRNA:vDNA ratios in LN, duodenal, and rectal biopsy tissues collected prior to dose 1 and 24 h after dose 6 for VES and vehicle control group animals (Fig. 7B). For the analyzed tissues, to avoid potential confounding effects of sample handling or preparation for analysis, we analyzed biopsy tissue specimens that were snap frozen immediately upon being obtained, but not otherwise handled or manipulated. There were no significant differences between VES-treated and control group animals at either time point in any of the three analyzed tissues (Fig. 7B). In general, cell-associated vRNA:vDNA ratios were comparably low in all three tissue types, though LN tissues (median: 0.3, range: 0.04–39 vRNA copies/vDNA copy) displayed greater variability and somewhat higher vRNA:vDNA levels than duodenum (median: 0.1, range: 0.03–1.0), rectum (median: 0.05, range: 0.006–1.7), or PBMC.

Lim and colleagues reported that following TLR7 agonist administration to SIV-infected macaques on cART, cell-associated vDNA levels declined in CD4+ memory T cells, particularly those collected from LN and gastrointestinal tissues (31). To evaluate potential changes in vDNA levels within our study animals, we quantified vDNA in PBMC, LN, duodenum, and rectum samples collected from VES and vehicle control animals prior to dose 1 and 24 h after dose 6. There were no significant differences in vDNA level between the pre-dose 1 and post-dose 6 samples for any of the sample types analyzed for either study group (Fig. 7C). Overall, vDNA levels were higher in PBMC and LN tissues compared with duodenal and rectal tissues, likely due to the greater proportional representation of CD4+ T cells within PBMC and LN tissues compared with gut tissues (Fig. 7C).

### Viral rebound following VES treatment

 In the study conducted by Lim and workers, one of three animals that received 19 cumulative doses of VES (GS-9620) at the same dose as that used in the present study (0.15 mg/kg) as well as one of three animals that received 19 cumulative doses of the related tool compound GS-986 (0.1 mg/kg) remained aviremic for over 2 years following cART cessation (31). We discontinued cART for the VES treatment group animals 26–27 weeks after the final dose of VES. Plasma viremia rebounded within 2 weeks of cART cessation in four of five animals, while rebound viremia was detected by week 4 in the fifth animal (Fig. S4). Off-cART PVLs broadly mirrored pre-cART PVLs. Animal H845A, which showed the highest pre-cART PVLs (Fig. 2), also had the highest peak rebound and setpoint PVLs of the group (Fig. S4). Conversely, animal G25A, which had the lowest pre-cART PVLs of the VES group animals (Fig. 2), had the longest period of aviremia before PVL rebound (4 weeks) with the lowest peak rebound PVL followed by subsequent virologic control to PVL levels below the assay quantification limit (<15 vRNA copies/mL) (Fig. S4).

## DISCUSSION

 The identification of agents that can induce the expression of persistent latent viral genomes during suppressive cART, particularly without counterproductive suppression of antiviral immune responses, constitutes a major component of the HIV cure research effort (57). While pharmacologic stimulation of TLR7 had long been viewed as a promising approach to boost either endogenous or vaccine-induced antiviral immune-responses, a study by Tsai and colleagues involving *ex vivo* VES treatment of PBMCs from people with HIV on suppressive cART suggested that TLR7 stimulation can also induce viral gene expression (58). This encouraging cell-culture result, which suggested that TLR7 stimulation might provide simultaneous induction of viral gene expression and immunologic boosting, provided the impetus for *in vivo* evaluation of TLR7 agonist administration in animal models of cART-suppressed AIDS virus infection. In an initial *in vivo* nonhuman primate study conducted by Lim and coworkers, SIVmac251-infected rhesus macaques were treated with daily cART beginning 65 days post-infection, during the early chronic phase of infection (31). After approximately 14 months of continuous cART, each animal received repeated oral administrations of the small molecule TLR7 agonist VES (GS-9620) or the related compound GS-986 once every 2 weeks. Using a PVL qRT-PCR assay with a nominal threshold sensitivity of 50 vRNA copies/mL, Lim and coworkers identified transient >10-fold PVL increases 24–48 h after each TLR7 agonist dose, indicative of induced viral gene expression and virion production from infected cells (31). These PVL increases were not observed after the first few TLR7 agonist doses, suggestive of an apparent “priming” mechanism underlying the virologic effects and underscoring the need to administer multiple TLR7 agonist doses to achieve viral reactivation (31).

 Despite these promising results reported by Lim et al., measurable viral induction following TLR7 agonist administration was not observed in a number of subsequent studies involving repeated administration of VES or GS-986, alone or in combination with other antiviral agents or immunizations, in SIV- or SHIV-infected macaques on cART (32–39). However, the experimental design and/or assays implemented for these studies may have limited their capacity to detect viral induction. While the Lim study animals were infected for ~9 weeks before receiving cART, most of the subsequent studies employed substantially earlier cART initiation (32–34, 36–39), often within the first 2 weeks of infection (32–34, 37–39). Earlier cART initiation may have limited the size of the inducible viral reservoir (16) and the resultant possible magnitude of the viral induction signal in these studies. Earlier cART initiation also may have led to qualitative differences in the infected cell population persisting during ART. Indeed, the duration between cART initiation and virologic suppression to below-threshold levels is often substantially longer in animals that initiate cART during the chronic phase of infection compared with those that start cART earlier (16, 17), suggestive of the establishment of a more “active” viral reservoir comprising cells that may be capable of producing more cumulative virus. Several of the studies that followed the Lim report also implemented nominally less sensitive PVL assays, with reported threshold sensitivities of 200-250 vRNA copies/mL (32, 33, 38), again potentially limiting their capacity to detect viral induction if it were to occur. Finally, though Lim et al. reported that PVL increases were typically transiently detected 24–48 h post-TLR7 agonist administration, most of the subsequent studies were not explicitly designed to assess viral induction and, thus, many did not evaluate specimens collected at these early time points (33, 35, 36, 38, 39, 52).

 In the present study, we sought to address the limitations of these prior studies and more faithfully capture the key features of the Lim et al. study. We hypothesized that measurable viral induction following TLR7 agonist administration in SIV-infected macaques on suppressive cART may depend upon quantitative and qualitative features of the residual viral reservoir, influenced by the timing of cART initiation, and upon the implementation of sensitive quantitative virologic assays. Following intravenous infection with SIVmac239M (43), we initiated daily cART in our study animals at 65 days post-infection, matching the timing of cART initiation utilized in the Lim study. Animals in our VES treatment group each received 6 doses of oral VES once every 2 weeks at 0.15 mg/kg, matching one of several doses, including a lower dose of 0.05 mg/kg, shown in the Lim study to induce transient PVL increases. To detect any changes in virus expression associated with VES administration, we employed a highly sensitive qRT-PCR assay with a threshold sensitivity of 15 vRNA copies/mL to quantify vRNA in peripheral venous plasma collected immediately prior to and at 24 and 48 h after each VES dose, corresponding to those time points that showed PVL increases following TLR7 agonist administration in the Lim study, as well as in hepatic portal venous plasma collected prior to VES dose 1 and then following each VES dose. We also employed highly sensitive qRT-PCR and qPCR methods to assess vRNA expression in PBMCs and tissues prior to and following VES administration.

Despite our use of a study design, specimen sampling scheme, and sensitive virologic methods explicitly intended to maximize the likelihood of detection of virus induction, and despite significant VES-induced immune modulation in our study animals, we did not observe any evidence of viral induction in response to VES administration via any of the measures we employed. Notably, each study animal had either episodic or regularly quantifiable PVLs during the VES-treatment phase of the study, indicating that the size and nature of established reservoirs and the sensitivity of the PVL assay employed were adequate to detect virus production under the conditions of our experiment. However, our data suggest that these quantifiable PVL fluctuations simply reflected normal biological variation rather than an effect of VES administration. We found no statistical evidence for PVL increases following VES administration, either when analyzing all 6 VES administrations or when analyzing only the final 3 VES administrations to allow for a potential “priming” effect like that suggested by the Lim study.

 While we cannot completely rule out the possibility that VES may have induced low-level viral expression that could not be detected by the methods we employed, our findings are broadly consistent with recent clinical evaluations of VES in PWH on suppressive cART (53, 54) in addition to a growing body of nonhuman primate studies involving VES administration to SIV- or SHIV-infected macaques on cART (32–39), all of which have failed to demonstrate evidence of TLR7 agonist-induced viral reactivation. That viral induction has not been evident in numerous studies conducted in nonhuman primates infected with different viruses via different routes nor in humans with HIV-1 suggests that viral inocula differences between the present study (SIVmac239M via the intravenous route) and the Lim study (SIVmac251 via the intrarectal route) are unlikely to explain the differences in our virologic results. Genetic comparisons between SIVmac239 and SIVmac251 have shown that they share as much genetic identity as can be found between HIV-1 genome sequences present in a single person with chronic HIV-1 (59, 60), and we are not aware of any studies that have shown that viral inoculation route fundamentally impacts the phenotype of viral reservoirs established in chronic infection. Because the *TLR7* gene is X-linked, TLR7 activity and responsiveness to TLR7 agonists can be influenced by sex and sex hormones, as well as age (61–73). While it is possible that demographic differences in the animal cohorts used in the Lim study and the present study may explain differences in the measured virologic responses to VES between the two studies, we note that all of our VES group animals showed clear immunomodulatory responses to VES treatment, indicative of effective TLR7 signaling. Although it remains unclear why Lim and colleagues observed striking PVL increases following TLR7 agonist administration, our data combined with those that we and others have previously reported suggest that TLR7 stimulation should not be viewed as an effective approach to potently induce lentiviral expression *in vivo*, either under a broad range of conditions or under a more narrow set of conditions similar to those employed in the Lim study, which might otherwise lend themselves to further investigation.

Although we were unable to demonstrate evident viral induction following VES administration, VES treatment resulted in clear immunomodulation in our study animals, including ISG induction in PBMCs, upregulated ISG protein expression in tissues, T and NK cell activation, and characteristic phenotypic changes in monocyte populations. Although we observed significant T cell activation in response to VES treatment, CD8+ T cells were substantially more responsive to VES-induced activation than were CD4+ T cells, which are the primary constituent of the RCVR during cART. Indeed, prior evaluations of CD4+ T cell activation following TLR7 agonist administration in macaques have yielded inconsistent results, with some studies showing significant CD4+ T cell activation (31–33, 36, 38, 39, 52), while others have shown no significant activation (34, 35). These discrepant findings may be explained by the fact that most prior studies have evaluated activation marker expression for the entire CD4+ T cell population, rather than separate memory subsets. Consistent with Lim et al., we show that while the CD4+ T_EM_ population displayed significant increases in activation following VES administration, indicated by the upregulation of both CD69 and CD38, CD4+ T_CM_ cells displayed no increases in CD69 expression and only modest increases in CD38 expression, while CD4+ T_N_ cells showed no apparent activation. This differential effect of VES on CD4+ T cell memory subsets suggests that the capacity of VES to induce meaningful viral reactivation may be limited. Prior studies have shown that while other CD4+ T cell subsets harbor persistent viral genomes during cART, CD4+ T_CM_ cells are the major cellular source of persistent viral genomes during therapy (74–76). The limited activation of this cellular subset may explain why we and others have not detected measurable viral induction following TLR7 stimulation. Our data also highlight that although CD69 expression has been the most commonly utilized marker to assess T cell activation following TLR7 agonist administration, CD38 may represent a more responsive marker for assessing the impact of TLR7 stimulation on T cells.

The present study was designed to evaluate VES-induced virus production in SIV-infected animals that initiated cART in early chronic infection. Although we also examined changes in vDNA and assessed viral rebound following VES administration, these experimental endpoints were not the focus of this work. In the original Lim study, vDNA levels declined in PBMCs and tissues following TLR7 agonist administration, and two of nine TLR7 agonist-treated macaques that discontinued cART maintained PVLs below 50 vRNA copies/mL for over 2 years after cART cessation (31). Notably, however, the animals in this phase of the Lim study received 19 cumulative doses of TLR7 agonist (31) compared with the 6 doses administered in the present study. A recent clinical evaluation of VES in cART-suppressed people with HIV-1 showed a modest delay in PVL rebound (5.1 weeks in VES-treated vs 4.1 weeks in controls) following 10 doses of VES (54). Intriguingly, while no changes in total vDNA were measured in the VES-treated clinical trial participants, a modest but significant decrease in intact viral genomes was measured. We confirmed that all five VES-treated animals that discontinued cART displayed relatively rapid viral rebound, detected within 4 weeks of cART interruption, consistent with viral rebound kinetics observed in prior studies involving SIV-infected macaques that received cART but were otherwise untreated (17, 45, 77). We, therefore, did not discontinue cART in our control group animals to assess whether there was any rebound delay associated with VES treatment as our study was not powered or intended to address this question, opting instead to maintain these animals on cART and repurpose them for another study not described here, conserving precious animal resources. Thus, it is unclear whether additional VES doses may have led to measurable vDNA reductions and/or viral rebound delays in our study animals.

Taken together, the available data suggest that the immunomodulatory effects of TLR7 agonists like VES, which have been consistently demonstrated across multiple studies, may enhance the clearance or control of viral reservoirs, but that the impact of this effect may be dependent upon the number of VES doses administered. Several studies conducted in humans and in macaques have provided direct and indirect evidence that TLR7 stimulation can enhance the antiviral activity of endogenous immune responses (34, 53, 54), vaccine-induced immune responses (32, 38, 39), and administered biologics (33, 37, 39, 52). However, given that limited expression of viral gene products during cART represents an inherent limitation for each of these potential viral targeting mechanisms, our findings argue for the evaluation of combinatorial approaches that include a potent viral reactivation strategy coupled with TLR7 stimulation to achieve meaningful RCVR reduction.

## SUPPLEMENTAL MATERIAL

Supplemental figures aac.01073-25-s0001.pdf Fig. S1 to S4.

Table S1 aac.01073-25-s0002.docx Animal group assignments.

ASM does not own the copyrights to Supplemental Material that may be linked to, or accessed through, an article. The authors have granted ASM a non-exclusive, world-wide license to publish the Supplemental Material files. Please contact the corresponding author directly for reuse.
