# Supplementary material for: The TLR7 agonist vesatolimod does not measurably induce SIV expression in macaques receiving combination antiretroviral therapy initiated during chronic infection
Source: Antimicrob Agents Chemother. 2025 Oct 7;69(11):e01073-25. doi: 10.1128/aac.01073-25 (PMC12587613; doi:10.1128/aac.01073-25)
Supplement: Supplemental figures — Fig. S1 to S4. [file aac.01073-25-s0001.pdf]

Supplementary Figure 1

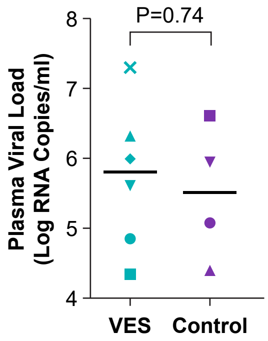

Supplementary Figure 2

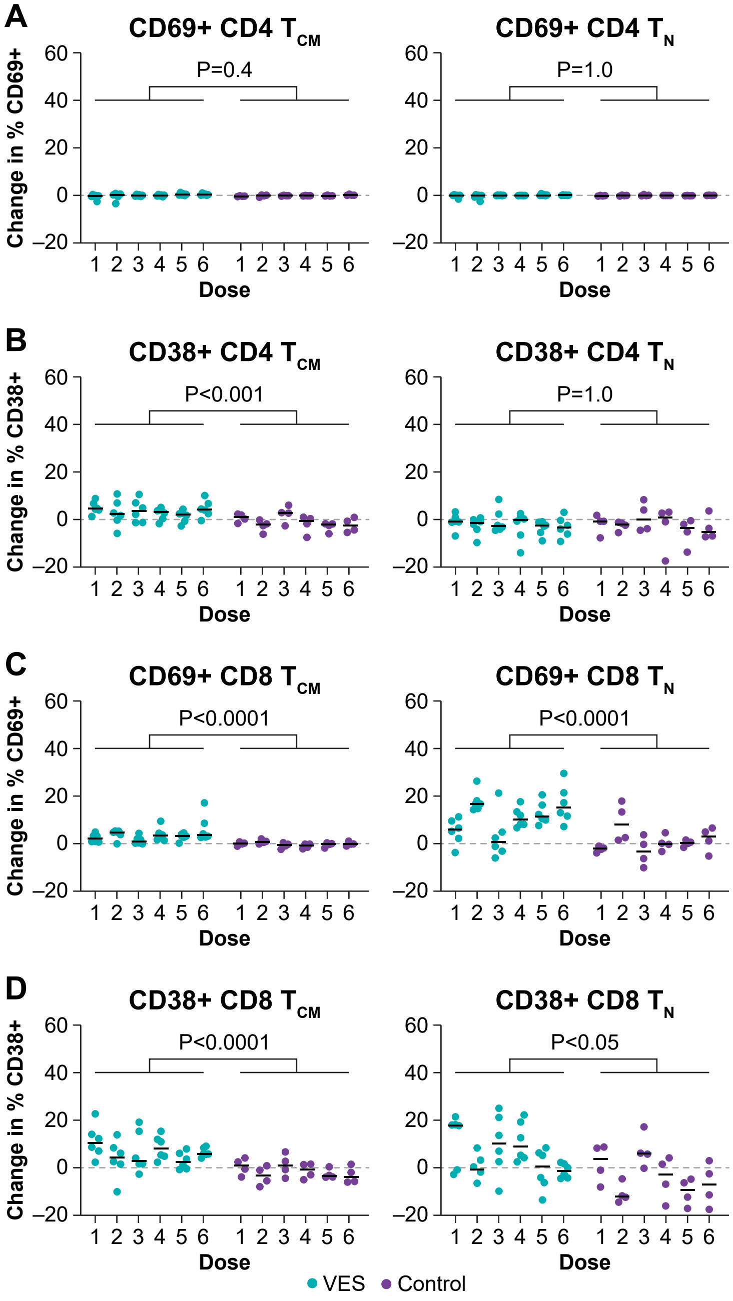

Supplementary Figure 3

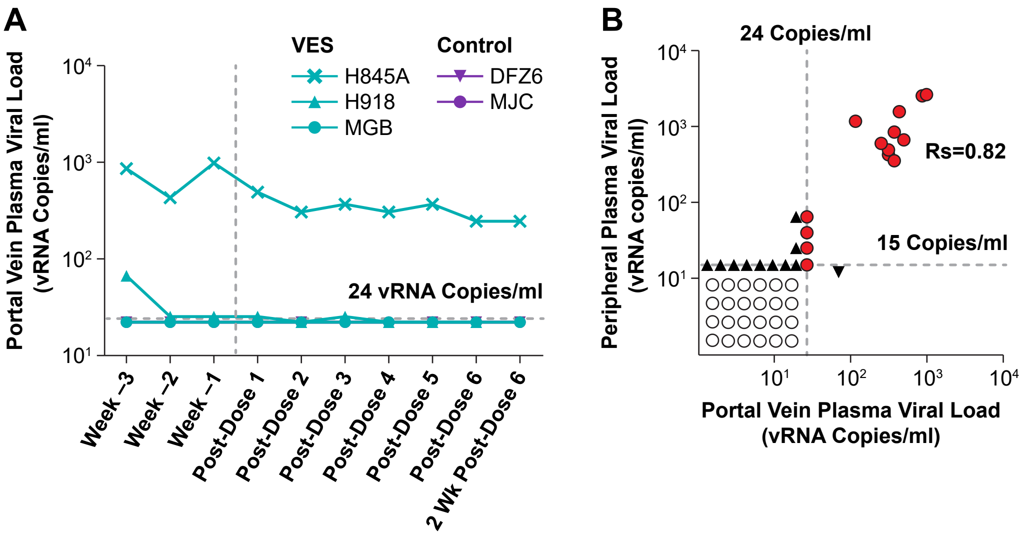

Supplementary Figure 4

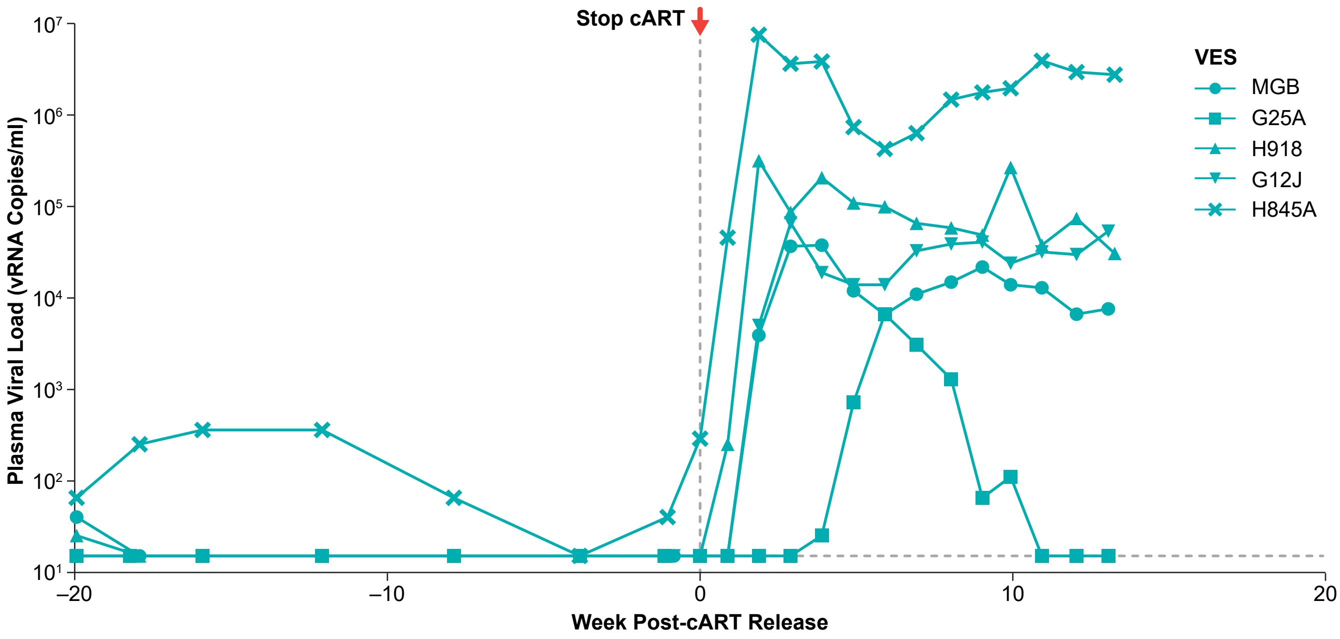

Supplementary Figure 1. Comparison of pre-cART plasma viral loads for study groups. SIV RNA measured in plasma on the day of cART initiation is shown for each animal assigned to the VES (teal) and vehicle control (purple) groups. There was no statistical difference in the pre-cART PVLs between the groups (two-sided *t*-test).

Supplementary Figure 2. Additional flow cytometric analysis of T cell activation markers in blood. Shown is the raw difference in the percentage of each indicated T cell memory population in blood expressing the indicated marker at 48 hours after each VES/vehicle dose compared with cells collected immediately prior to the corresponding VES/vehicle dose for each animal (% of cells expressing marker following dose minus % of cells expressing marker prior to dose). Following VES/vehicle administration, there were no significant differences between VES treated control animals in the change in the percentage of CD4+ T_{CM} or T_N cells expressing CD69 **(A)** or CD4+ T_N cells expressing CD38 **(B)**, but there were significant increases in the percentage of CD4+ T_{CM} cells expressing CD38 **(B)**, and CD8+ T_{EM} and T_N cells expressing CD69 **(C)** and CD38 **(D)** (unpaired Wilcoxon rank sum tests).

Supplementary Figure 3. Plasma viral loads in hepatic portal vein blood. (A) Longitudinal SIV RNA quantification was performed on plasma collected from the hepatic portal vein of the indicated animals. Plasma was extracted from blood samples collected for 3 weeks prior to the first VES/vehicle dose and then at 24 hours after each dose and two weeks after the final dose. For animal H918, the post-dose samples for doses 3 and 4 were collected at 48 hours post-dose rather than 24 hours post-dose. No blood sample was collected for animal DFZ6 following dose 4. The horizontal dashed line represents the threshold sensitivity for the plasma viral load assay utilized (24 vRNA copies/ml). (**B)** Correlation analysis of plasma viral loads measured in peripheral blood samples and hepatic portal vein blood samples collected at the same time points. Open circles, time points for which SIV RNA was not detected in either hepatic portal vein plasma or

peripheral blood plasma; triangles, time points for which SIV RNA was detected and quantified in peripheral blood plasma but was below detection limits in portal vein plasma; inverted triangles, time points for which SIV RNA was detected and quantified in portal vein plasma but was below detection limits in peripheral blood plasma; red circles, time points for which SIV RNA was detected and quantified in both hepatic portal vein plasma and peripheral blood plasma. A Spearman rank correlation between within-time-point within-subject pairs was performed for those time points for which at least one of the two values was above threshold (N = 25; Rs = 0.82).

Supplementary Figure 4. Off-cART rebound plasma viral loads in VES group animals. Combination ART was discontinued for the indicated VES group animals. Shown are longitudinal PVL measurements for these animals for the time period spanning 20 weeks prior to cART discontinuation through 14 weeks after cART discontinuation. The threshold sensitivity for the PVL assay utilized (15 vRNA copies/ml) is shown as a horizontal dashed line.
