# Supplementary material for: The TLR7 agonist vesatolimod does not measurably induce SIV expression in macaques receiving combination antiretroviral therapy initiated during chronic infection
Source: Antimicrob Agents Chemother. 2025 Oct 7;69(11):e01073-25. doi: 10.1128/aac.01073-25 (PMC12587613; doi:10.1128/aac.01073-25)
Supplement: Table S1 — Animal group assignments. [file aac.01073-25-s0002.docx]

| **Supplementary Table 1. Animal Group Assignments** | | | | |
| --- | --- | --- | --- | --- |
| **Group** | **ID** | **Sex** | **Mamu-A*01** | **Pre-cART Plasma Viral Load (vRNA Copies/ml)** |
| VES | MGB | F | + | 7.1 × 10^4^ |
| VES | G25A | F | + | 2.2 × 10^4^ |
| VES | H918 | F | + | 2.1 × 10^6^ |
| VES | G12J | F | - | 4.1 × 10^5^ |
| VES | 12M153 | M | + | 9.9 × 10^5^ |
| VES | H845A | M | - | 2.0 × 10^7^ |
| Control | MJC | F | + | 1.2 × 10^5^ |
| Control | MLG | F | - | 4.1 × 10^6^ |
| Control | DFZ8 | M | + | 2.5 × 10^4^ |
| Control | DFZ6 | M | - | 8.9 × 10^5^ |
